# Discovery and functional analysis of a novel ALPK1 variant in ROSAH syndrome

**DOI:** 10.1098/rsob.240260

**Published:** 2024-12-04

**Authors:** Tom Snelling, Leo O. Garnotel, Isabelle Jeru, Maud Tusseau, Laurence Cuisset, Antoinette Perlat, Geoffrey Minard, Thibaut Benquey, Yann Maucourant, Nicola T. Wood, Philip Cohen, Alban Ziegler

**Affiliations:** ^1^ MRC Protein Phosphorylation and Ubiquitylation Unit, School of Life Sciences, University of Dundee, Dundee DD1 5EH, UK; ^2^ Department of Ophthalmology, University Hospital of Reims, Reims, France; ^3^ Department of Medical Genetics, Sorbonne Université, Assistance Publique-Hôpitaux de Paris, Hôpital Pitié-Salpêtrière, Paris, France; ^4^ Centre International de Recherche en Infectiologie, Inserm, U1111, University Claude Bernard, Lyon 1, UMR5308, ENS de Lyon, Lyon, France; ^5^ Hospices Civils de Lyon, Department of Medical Genetics, University Hospital of Lyon, Lyon, France; ^6^ Université Paris Cité, Service de Médecine Génomique des Maladies de Système et D'Organe, Hôpital Cochin, Assistance Publique-Hôpitaux de Paris, Paris, France; ^7^ Department of Internal Medicine and Clinical Immunology, Pontchaillou Hospital, Rennes, France; ^8^ Department of Internal Medicine and Clinical Immunology, University Hospital of Reims, Reims, France; ^9^ Laboratory Eurofins Biomnis, Lyon, France; ^10^ Department of Ophthalmology, Pontchaillou Hospital, Rennes, France; ^11^ Department of Medical Genetics, University Hospital of Reims, Reims, France; ^12^ Department of Medical Genetics, University Hospital of Toulouse, Toulouse, France

**Keywords:** ADP-heptose, ALPK1, nucleotide sugar, UDP-mannose, ROSAH, TIFA

## Abstract

Retinal dystrophy, optic nerve oedema, splenomegaly, anhidrosis and migraine headache (ROSAH) syndrome is an autosomal dominant disorder and to date is known to be caused by either the Thr237Met or Tyr254Cys variant in the protein kinase ALPK1. Here, we identify a family in which ROSAH syndrome is caused by a novel variant in which Ser277 is changed to Phe. All six patients examined display ocular inflammation and optic nerve elevation, four have retinal degeneration and four are registered blind. In contrast to wild-type ALPK1, which is activated specifically by bacterial ADP-heptose, ALPK1[Ser277Phe] is also activated by the human metabolites UDP-mannose and ADP-ribose and more strongly than the most frequent ROSAH-causing variant (ALPK1[Thr237Met]) but, unlike ALPK1[Thr237Met], ALPK1[Ser277Phe] is also activated by GDP-mannose. These observations can explain why ALPK1 variants causing ROSAH syndrome display constitutive activity in human cells. The side chains of Ser277 and Tyr254 interact in the crystal structure of ALPK1, but mutational analysis established that it is not the loss of this hydrogen bond between Ser277 and Tyr254 that alters the specificity of the ADP-heptose-binding pocket in the Ser277Phe and Tyr254Cys variants. The characterization of ALPK1 variants that cause ROSAH syndrome suggests ways in which drugs that selectively inhibit these disease-causing variants may be developed.

## 1. Introduction

ALPK1 (alpha-kinase 1) is a key component of an innate immune signalling pathway that is activated by the bacterial nucleotide sugars ADP-d-glycero-β-d-manno-heptose (ADP-d,d-heptose) and ADP-l,d-heptose (hereafter ADP-heptose) [1]. The binding of either of these ADP-heptoses within the N-terminal domain of ALPK1 activates the C-terminal catalytic kinase domain, enabling ALPK1 to phosphorylate TIFA (TRAF-interacting protein with forkhead-associated domain) [1]. This leads to the polymerization of TIFA and the consequent formation of a signalling complex that activates the transcription factors NF-κB (nuclear factor kappa-light-chain-enhancer of activated B cells) and AP-1 (activator protein 1) [1–4].

Retinal dystrophy, optic nerve oedema, splenomegaly, anhidrosis and migraine headache (ROSAH) syndrome is an autosomal dominant disease caused by pathogenic variants in ALPK1 [5]. Patients with ROSAH syndrome usually present in the clinic with failing eyesight but, beyond this, the precise phenotype varies and can additionally include autoinflammatory conditions, such as arthritis [6]. In all but one patient with ROSAH so far identified, the variant Thr237Met of ALPK1 was found, the exception being a single patient with a Tyr254Cys variant [7]. Thr237 and Tyr254 are located within the N-terminal ADP-heptose binding domain of ALPK1, but only Thr237 forms a direct interaction with ADP-heptose itself [1].

The overexpression of ALPK1[Thr237Met] or ALPK1[Tyr254Cys] in HEK293 (human embryonic kidney 293) cells activates NF-κB/AP-1-dependent gene transcription in the absence of ADP-heptose in the cell culture medium, implying that these mutated proteins are activated by an ADP-heptose-independent mechanism in cells [4]. However, when immunoprecipitated from cell extracts and assayed for kinase activity, ALPK1[Thr237Met] is devoid of kinase activity in the absence of ADP-heptose, similar to the wild-type (WT) enzyme [4]. These observations led to the unexpected finding that, in contrast to WT ALPK1, ALPK1[Thr237Met] can also be activated by the human nucleotide sugars UDP-mannose and ADP-ribose in cell-free assays [4]. This suggested that activation of ALPK1[Thr237Met] by one or more human sugar nucleotides may cause chronic activation of ALPK1 in cells and underlie autoinflammation in ROSAH syndrome. In contrast to ALPK1[Thr237Met], the ALPK1[Tyr254Cys] mutant was devoid of activity when assayed in this way, even in the presence of ADP-heptose, indicating that it is unstable when removed from cells. For this reason, it has not been possible to determine whether the ALPK1[Tyr254Cys] mutant is activated by UDP-mannose and/or ADP-ribose.

So far, 67 patients with ROSAH syndrome from 29 unrelated families and carrying only two different missense variants have been reported [5–10]. However, as the first case was only described in 2019, and all of the patients described so far come from only seven countries, the condition is probably underdiagnosed and many more patients with the disease are likely to be identified. Here, we report on the identification of an additional family with ROSAH syndrome caused by a new Ser277Phe variant in ALPK1 and characterize its effect on ALPK1 activity.

## 2. Material and methods

### 2.1. Genetic studies

Diagnostic laboratories performed genetic analyses on genomic blood DNA extracted from peripheral blood leucocytes using standard procedures. Genome sequencing for individuals II1 and III1 was performed at the SeqOIA laboratory (LBMS SeqOIA, Paris, France). FASTQ files were obtained from the bcl2fastq demultiplexing tool (v. 2.20.0.422, Illumina) and aligned to the GRCh38.92 genomic reference using BWA-MEM (v. 0.7.15). Haplotype Caller (v. 4.1.7.0) was used to call single nucleotide variants and delins (<50 bp); variants were annotated by SNPEff (v. 4.3t). Structural variants were detected by ClinSV (v. 1.0.1) and WiseCondor (v. 1.2.4). The resulting structural variants were then annotated with AnnotSV (v. 3.0.7) [11]. In-house software (GLEAVES, v. 3.3.23) was used for the variant interpretation and reporting. Further details are available on the LBMS SeqOIA platform (https://laboratoire-seqoia.fr/). Exome sequencing for individual II2 was performed at Biomnis laboratory (Lyon, France) using a Human Exome 2.0 Plus Comprehensive Exome library and a Novaseq 6000 sequencer.

### 2.2. Antibodies

Antibodies recognizing GAPDH (2118) and anti-rabbit IgG (7074) were from Cell Signalling Technology and an antibody recognizing FLAG (#F3165) was from Sigma-Aldrich.

### 2.3. DNA constructs

The following DNA plasmids encoding FLAG-tagged WT or variant forms of ALPK1 with expression under the control of the cytomegalovirus promoter were made by Medical Research Council Reagents and Services, Medical Research Council Protein Phosphorylation and Ubiquitylation Unit, University of Dundee and assigned unique identifiers (listed in parentheses); these constructs are available on request (mrcppureagents.dundee.ac.uk): WT (DU65668), R150A (DU71740), R150A/T237M (DU71743), R150A/Y254C (DU71741), R150A/S277F (DU71954), T237A (DU71986), T237M (DU65723), Y254C (DU71685), Y254F (DU71987), S277A (DU71988), S277F (DU71952) and S277F/K1067M (DU71956).

### 2.4. Nucleotide sugars

The sources of nucleotide sugars are as described [4], except that the ADP-heptose used in this study was from Invivogen (tlrl-adph-l). All nucleotide sugars were prepared as 1 mM stock solutions in phosphate-buffered saline (PBS).

### 2.5. Transfection of HEK293-blue cells in 96-well format and measurement of NF-κB/AP-1 gene transcription

A detailed protocol for this procedure has been published [12]. Briefly, cells (60 000) in 0.1 ml of Dulbecco’s modified Eagle medium containing 10% (v/v) heat-inactivated foetal bovine serum were ‘reverse’ transfected in a 96-well plate using 1 µl of lipofectamine 2000 (ThermoFisher, 11668019) and 0.4 µg of plasmid DNA. After 24 h, the culture medium was aspirated and replaced by 75 µl of Dulbecco’s modified Eagle medium containing 10% (v/v) heat-inactivated foetal bovine serum, 100 U ml^−1^ penicillin and 0.1 mg ml^−1^ streptomycin with or without 5 μM ADP-heptose. This culture medium was then collected after a further 24 h, and 50 µl was incubated with 150 µl of QUANTI-blue solution (Invivogen, rep-qbs) in a new 96-well plate. The plate was incubated for 30 min at room temperature and the absorbance at 645 nm was measured using a microplate reader. For detection of ALPK1 expression by immunoblotting, the cells were lysed in 30 µl sodium dodecylsulfate (SDS) sample buffer (Millipore, 70607) supplemented with 0.5% (v/v) benzonase endonuclease (Sigma-Aldrich, E1014), 1 mM MgCl_2_ and protease inhibitor cocktail (Roche, 11836170001).

### 2.6. Cell-free ALPK1 phosphorylation assays

A detailed protocol for this procedure has been published [13]. Briefly, plasmid DNA (60 µg) encoding WT or mutant ALPK1 was transfected into 15 cm dishes of ALPK1 KO HEK293-Blue cells using 150 µl of lipofectamine 2000. After 24 h, the cells were washed twice with PBS and scraped in 1 ml ice-cold lysis buffer containing 50 mM Tris–HCl (pH 7.5), 1 mM EDTA, 1 mM EGTA, 1% (v/v) Triton X-100, 2 mM dithiothreitol (DTT) and 270 mM sucrose supplemented with protease inhibitor cocktail (Roche, 11836170001). Cell lysates were clarified by centrifugation for 20 min at 20 000×*g* at 4°C and the supernatants (cell extracts) were transferred to 1.5 ml microcentrifuge tubes. Cell extract protein (0.05 mg) containing WT FLAG-ALPK1 or FLAG-ALPK1 mutants (normalized for ALPK1 expression) were incubated for 1 h at 4°C on a rotating wheel with 15 μl of packed anti-FLAG M2 affinity gel (Sigma-Aldrich, A2220), which had been washed twice with lysis buffer prior to use. After centrifugation for 30 s at 1000×*g* at 4°C, the supernatant was discarded and the pelleted gel was washed three times with 50 mM Tris–HCl (pH 7.5), 2 mM DTT, 0.1% Triton X-100 (Buffer A) containing 500 mM NaCl, twice with Buffer A and once with 50 mM Tris–HCl (pH 7.5), 2 mM DTT, 0.1 mM EGTA and 10 mM magnesium acetate (Buffer B). The immunoprecipitated FLAG-ALPK1 was then incubated at 30°C in 25 µl of Buffer B containing 2.1 μM glutathione-*S*-transferase (GST)-TIFA (dialysed against 50 mM Tris–HCl (pH 7.5), 2 mM DTT) and 0.1 mM [γ-^32^P]ATP (specific radioactivity 500 cpm pmol^−1^). The reactions were initiated by the addition of the [γ-^32^P]ATP and terminated after 30 min by the addition of lithium dodecylsulfate sample buffer (ThermoFisher, P0008) containing 2.5% (v/v) 2-mercaptoethanol and heated for 5 min at 75°C. The FLAG resin was pelleted by centrifugation for 30 s at 13 000×*g* and the supernatant was subjected to SDS-PAGE. After staining for 30 min with InstantBlue Protein Stain (Abcam, ab119211) and destaining for 16 h in water with frequent changes, the bands corresponding to GST-TIFA were excised, and the incorporation of ^32^P-radioactivity analysed by Cerenkov counting. The cpm values obtained were converted to pmol of phosphate using the specific radioactivity of the ATP used.

### 2.7. Other procedures

All other methods, including the origin and culturing of cells, have been described elsewhere [4].

## 3. Results and discussion

### 3.1. Clinical investigation of a family harbouring the symptoms of ROSAH syndrome

**Figure 1.**
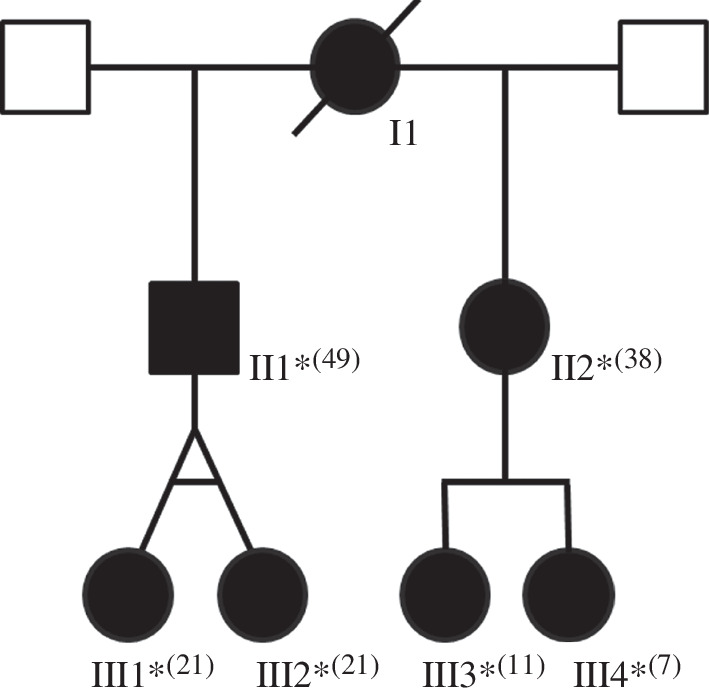
Family expressing the ALPK1[Ser277Phe] variant. Individual II1 and individual II2 have the same mother but different fathers. The daughters of individual II1 are monozygous twins. The patients that have been analysed clinically are marked by an asterisk, and the ages at which they were last evaluated are given in parentheses.

Individual I1 is the mother of individuals II1 and II2 (figure 1). She died from breast cancer but had a history of progressive visual impairment from her 20s and was registered blind in her 40s. However, her DNA was not available for sequencing. The clinical symptoms of her son (individual II1) and daughter (individual II2) and their children are summarized in table 1.

**Table 1.** Disease characteristics of patients heterozygous for the ALPK1[Ser277Phe] variant likely to be pathogenic. *The diagnosis of hypohidrosis was based on patient feedback. **Patient II2 reported numerous episodes of macroscopic haematuria, most often associated with fever. ***ND (not determined). ****Cutaneous allergy to tocilizumab necessitated a switch to baricitinib, an inhibitor of JAK kinases.

	individual						
feature	II1	II2	III1	III2	III3	III4	total
age at last evaluation	**49**	**38**	**21**	**21**	**11**	**7**	
ocular inflammation	**++**	**++**	**+**	**+**	**+**	**+**	**6/6**
retinal degeneration	**+**	**+**	**+**	**+**	**−**	**−**	**4/6**
optic nerve elevation	**+**	**+**	**+**	**+**	**+**	**+**	**6/6**
hypohidrosis*	**+**	**+**	**+**	**+**	**−**	**−**	**4/6**
headache	**−**	**−**	**+**	**+**	**−**	**−**	**2/6**
joint involvement	**+**	**+**	**+**	**+**	**−**	**+**	**5/6**
splenomegaly	**−**	**+**	**−**	**−**	**−**	**−**	**1/6**
recurrent fever**	**−**	**+**	**−**	**−**	**−**	**−**	**1/6**
haematuria**	**−**	**+**	**−**	**−**	**−**	**−**	**1/6**
dry mouth	−	+	−	−	−	**−**	**1/6**
enamel defects/multiple dental caries	**−**	**+**	**−**	**−**	**−**	**−**	**1/6**
episodic abdominal pain	**−**	**−**	**−**	**−**	**−**	**−**	**0/6**
episodic malaise	**−**	**−**	**−**	**−**	**−**	**−**	**0/6**
positive response to tocilizumab (anti-IL-6)	ND***	+	+	+ ****	ND***	ND***	**3/3**

Individual II1 is 49, the father of individuals III1 and III2 and the maternal half-brother of individual II2 (figure 1). He has intermediate and posterior uveitis, associating hyalitis, bilateral papilloedema, major macular oedema with pre-retinal neo-vessels, and was registered blind in his 40s. He also has painful arthralgia of the hands and knees.

Individual II2 (figure 1) is 38 and originally presented with bilateral eye pain and visual loss. Her personal medical history was marked by unclear episodes of recurrent ocular inflammation since the age of 20. The initial best corrected visual acuity of individual II2 was 20/32 (both eyes) and an optic disc oedema was present. A bilateral retinal nerve fibre layer (RNFL) thickening was confirmed on optical coherence tomography (OCT), and macular cysts in inner retinal layers were found (electronic supplementary material, figures S1 and S2). Like her half-brother, she has arthralgia of the hands consecutive to deforming arthritis. Her symptoms are presented in table 1.

Initial management with topical corticosteroid drops resolved anterior inflammation, with no effect on posterior signs. She was lost on follow-up and presented a year after with a recurrence of bilateral anterior inflammation. Systemic corticosteroids (1 mg kg^−1^) were then introduced and tapered with a good efficacy on macular and optic disc thickening up to 10 mg (electronic supplementary material, figure S3). Given this partial response, an anti-IL-6 therapy (tocilizumab) was introduced to further reduce inflammation and thickening of the posterior retina and optic disc. A four-month follow-up examination confirmed that macular and optic disc thickening had decreased, and visual acuity had improved.

Individuals III1 and III2 (figure 1) are 21-year-old female monozygotic twins and present with identical symptoms. They were born prematurely at 34 weeks of gestation and have had delays in learning and speech development. Visual acuity at the age of 10 was normal. At the age of 12, during an evaluation for decreased visual acuity, a diagnosis of bilateral macular and papillary oedema with pre-retinal neovascularization was made, along with polyarthralgia and finger deformities characterized by broadening of the proximal interphalangeal joints without active synovitis. At the age of 14, the visual acuity of individual III1 was 1.2/10 P12 for the right eye and 1.4/10 P12 for the left eye; that of individual III2 was 9/10 P2 for the right eye and 0.8/10 P28 for the left eye. They also have autoimmune hypothyroidism, which was diagnosed at the age of 12 in both patients.

These two patients were initially treated for macular oedema with systemic corticosteroids and local steroid injections. Systemic corticosteroids did not reduce the macular oedema, but iterative local injections of corticosteroids were successful in relieving the uveitis. Methotrexate was introduced to reduce the use of corticosteroids, followed by anti-TNF therapies (adalimumab and then infliximab). Intravenous tocilizumab (anti-IL-6) was started in 2021, which was effective in reducing uveitis when used in combination with local dexamethasone injections, transitioning to subcutaneous injection for individual III2. Individual III2 developed a cutaneous allergy to tocilizumab, necessitating a switch to a JAK kinase inhibitor (baricitinib). In addition to this systemic treatment, injections of fluocinolone acetonide implant for individual III1 and dexamethasone implants for individual III2 were performed once or twice a year. Multiple dexamethasone injections triggered bilateral cataracts in both patients, which were operated on between 2020 and 2022.

Pre-retinal haemorrhages associated with the neo-vessels were also noted, for which several laser sessions and intravitreal anti-vascular endothelial growth factor injections were performed. The last haemorrhage was identified at the age of 16 in the two patients, with no recurrence since.

The latest acuities at the age of 21 were 2/10th P14 in both eyes for individual III1 and 1.6/10th P8 on the right eye and 1.4/10th P16 on the left for individual III2. They are therefore registered blind. However, their intraocular pressures have always been normal.

Individuals III3 and III4 are the daughters of individual II2 (figure 1). At the last evaluation at age 11, III3 had a normal fundus, but macular and RNFL OCTs showed a discrete thickening of RNFL and internal retinal layers. Individual III4 at the last evaluation was age 7 and an optic nerve head oedema was clearly visible on the fundus. Her macular and RNFL OCTs also showed a thickening of RNFL and internal retinal layers. In both cases, oedema was present along the path of RNFL and retinal vasculature.

The phenotypic variability in this family is high, especially concerning the severity of the visual impairment with four individuals (I1, II1, III1 and III2) registered blind, while the other three (II2, III3 and III4) have none to mild visual impairment at present. Notably, this variability is not only related to age as individual II2 with a mild visual impairment is much older than her nieces (individuals III1 and III2). This less severe phenotype might be related to a genetic contribution from the father of individual II2.

### 3.2. Characterization of a novel *ALPK1* variant

Genome sequencing was performed on the DNA of subjects II1 and III1 and exome sequencing on subject II2. This led to the identification of these affected members having a heterozygous missense variant in exon 10 of *ALPK1*: c.830C>T; p.Ser277Phe (transcript NM_025144.4). The segregation of the variant was confirmed by Sanger sequencing in subjects III2, III3 and III4. Notably, we did not identify any alternative molecular aetiology compatible with the disease phenotype in any of these three patients. Several additional lines of evidence supported the causal role of this variant in the disease phenotype. First, the variant was absent from databases reporting variants from the general population (gnomAD v. 4.1.0), as well as from ClinVar, a database that aggregates information about genomic variations and their relationship to human diseases. Second, Ser277 is an amino acid that is strongly conserved in all major vertebrate lineages. Third, the familial segregation and clinical overlap with other variants causing ROSAH syndrome suggested that the p.Ser277Phe variant was likely to be pathogenic. In the sections that follow, we study the functional impact of the ALPK1[Ser277Phe] variant on ALPK1 activity which has further established the pathogenicity of the variant. We have, therefore, deposited the p.Ser277Phe to ClinVar as a pathogenic variant (accession no. SCV005375334).

### 3.3. Effect of the ALPK1[Ser277Phe] mutation on NF-κB/AP-1-dependent gene transcription in HEK293 cells

**Figure 2.**
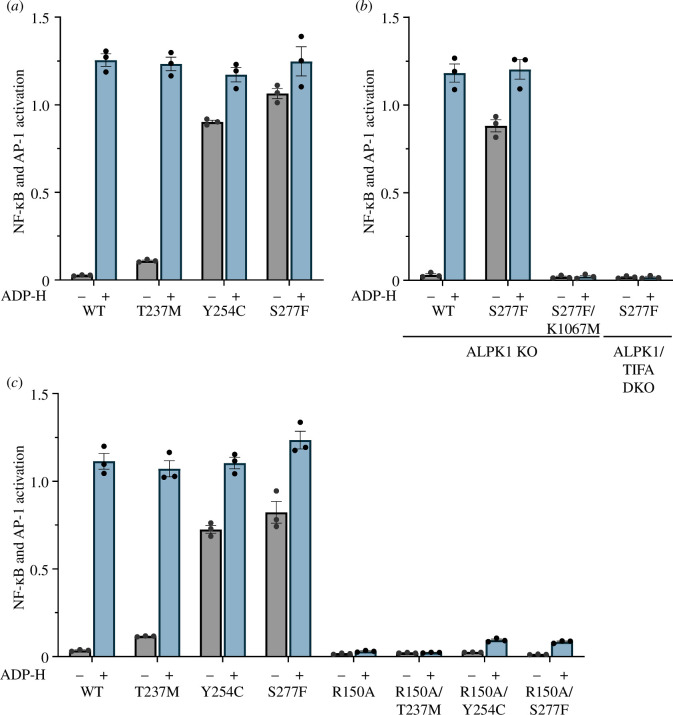
The ALPK1[Ser277Phe] mutant stimulates NF-κB/AP-1-dependent gene transcription in the absence of ADP-heptose. (*a*) ALPK1 KO cells were transfected with plasmids encoding WT ALPK1 or the indicated ALPK1 mutants. Twenty-four hours later, cells were incubated with (blue bars) or without (grey bars) 5 μM ADP-heptose (ADP-H) and NF-κB/AP-1-dependent gene transcription was measured after another 24 h (see §2). (*b*) ALPK1 KO or ALPK1/TIFA double KO (DKO) cells were transfected with the plasmids indicated and analysed as in (*a*). (*c*) As in (*a*) but using plasmids in which each ROSAH-causing mutant was combined with Arg150Ala, which disrupts the ADP-heptose binding. (*a–c*) Results are shown as the mean of an experiment performed in triplicate, where individual values are denoted by filled circles and the error bars indicate plus and minus one standard error of the mean. Similar results were obtained in two additional, independent experiments.

We initially compared gene transcription induced by ALPK1 mutants after re-expressing them in ALPK1 knockout (KO) HEK293 cells, which contain a synthetic gene encoding secreted embryonic alkaline phosphatase under the control of NF-κB and AP-1 promoters. In this assay, we found that gene transcription induced by ADP-heptose was similar in cells expressing WT ALPK1, ALPK1[Ser277Phe], ALPK1[Thr237Met] or ALPK1[Tyr254Cys] (figure 2*a*). When ADP-heptose was omitted, gene transcription was negligible in cells expressing WT ALPK1, but was still observed in cells expressing ALPK1[Ser277Phe], ALPK1[Thr237Met] or ALPK1[Tyr254Cys] (figure 2*a*). The extent of gene transcription induced by ALPK1[Ser277Phe] and ALPK1[Tyr254Cys] was greater than that induced by the ALPK1[Thr237Met] mutant (figure 2*a*). The level of expression of the ALPK1[Ser277Phe] mutant in transfected ALPK1 KO cells was similar to that of WT ALPK1 (electronic supplementary material, figure S4).

The activation of NF-κB/AP-1-dependent gene transcription by ALPK1[Ser277Phe] did not occur in TIFA KO cells, or when the Ser277Phe mutation was combined with the Lys1067Met mutation (figure 2*b*). The latter mutation is located within the kinase domain and disrupts ATP binding at the catalytic site [1]. Thus, the kinase activity of ALPK1 and the expression of TIFA are both required for ALPK1[Ser277Phe] to induce gene transcription, irrespective of whether ADP-heptose is present. These results are similar to those obtained previously with the ALPK1[Thr237Met] and ALPK1[Tyr254Cys] variants and this suggested that, like these variants [4], ALPK1[Ser277Phe] might be activated in cells by human nucleotide sugars.

To investigate if ALPK1[Ser277Phe] was active in cells because it was being activated by another molecule that binds to the same site as ADP-heptose, we disrupted the ADP-heptose binding site. Arg150 is an amino acid residue that has been shown to be critical for ADP-heptose binding because it forms electrostatic interactions with the negatively charged phosphate groups of ADP-heptose within the ADP-heptose-binding site [1]. We found that mutation of Arg150 to Ala not only abolished (WT ALPK1 or ALPK1[Thr237Met]) or greatly reduced (ALPK1[Tyr254Cys] or ALPK1[Ser277Phe]) gene transcription in the presence of ADP-heptose, but also activity in the absence of this nucleotide sugar (figure 2*c*). This was not caused by the failure of the ‘double mutant’ proteins to express, although expression was reduced relative to the ‘single’ mutants (electronic supplementary material, figure S4). The finding that the Arg150Ala/Ser277Phe double mutant of ALPK1 failed to stimulate gene transcription supported the hypothesis that ALPK1[Ser277Phe] was being activated in cells by another molecule engaging the ADP-heptose binding site and led us to study the effects of human nucleotide sugars.

### 3.4. Effect of the ALPK1[Ser277Phe] mutation on ALPK1 activity in cell-free kinase assays

**Figure 3.**
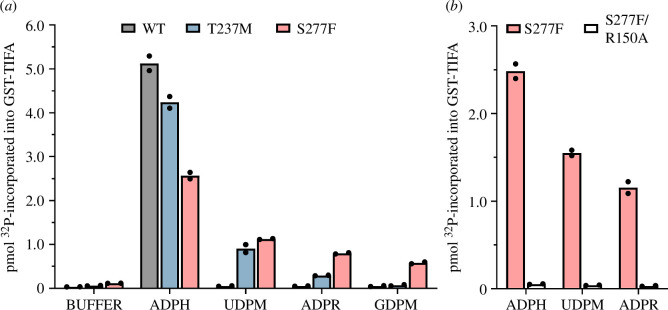
ALPK1[Ser277Phe] is activated by mammalian nucleotide sugars in cell-free phosphorylation assays. (*a*) FLAG-tagged WT ALPK1 (WT, grey bars), ALPK1[Thr237Met] (T237M, blue bars) or ALPK1[Ser277Phe] (S277F, pink bars) were immunoprecipitated from cell extracts and assayed for 30 min in the absence or presence of 5 µM ADP-heptose (ADPH) or 100 µM UDP-α-d-mannose (UDPM), ADP-d-ribose (ADPR) or GDP-α-d-mannose (GDPM), and the amount of phosphate (in pmol) incorporated into GST-TIFA plotted (see §2). (*b*) As in (*a*), except comparing ALPK1[Ser277Phe] (S277F, pink bars) and ALPK1[Ser277Phe/Arg150Ala] (S277F/R150A, unfilled bars). (*a,b*) Two independent experiments were performed, each in duplicate. The results from each individual experiment were averaged and are represented by the filled circles. The bar heights reflect the mean of these two values.

To identify human metabolites that might activate ALPK1[Ser277Phe], we assayed its activity in cell-free kinase assays. We found that, similar to ALPK1[Thr237Met], ALPK1[Ser277Phe] was activated by UDP-α-d-mannose and ADP-d-ribose, but more strongly than ALPK1[Thr237Met] (figure 3*a*). Moreover, the activation of ALPK1[Ser277Phe] by UDP-α-d-mannose and ADP-d-ribose was prevented by combination with the Arg150Ala mutation (figure 3*b*), indicating that the mammalian nucleotide sugars activate ALPK1[Ser277Phe] by binding to the ADP-heptose binding site. Interestingly, and unlike ALPK1[Thr237Met] or WT ALPK1, ALPK1[Ser277Phe] was additionally activated by GDP-α-d-mannose (figure 3*a*). The activity of the ALPK1[Ser277Phe] mutant in the presence of ADP-heptose was consistently lower than that of ALPK1[Thr237Met] in the presence of ADP-heptose which, in turn, was lower than that of WT ALPK1 (figure 3*a*).

### 3.5. The roles of Ser277, Tyr254 and Thr237 in ADP-heptose binding

**Figure 4.**
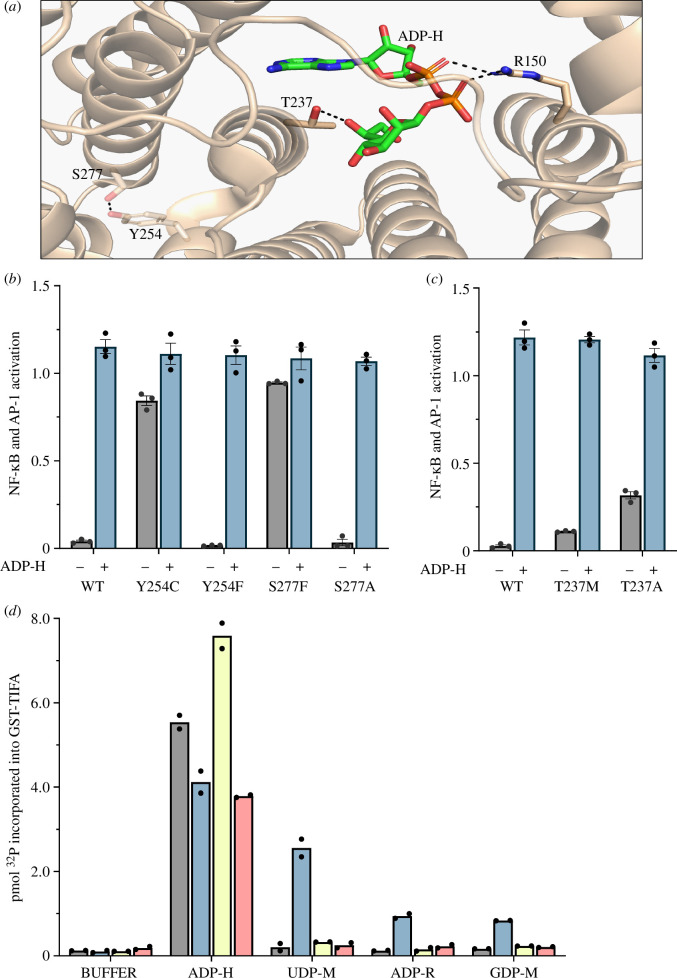
Interactions between Ser277 and Tyr254, and between Thr237 and ADP-heptose, within the ADP-heptose binding domain of ALPK1. (*a*) Location of Ser277, Tyr254, Thr237 and Arg150 in the ADP-heptose (ADP-H) binding domain of ALPK1 (PDB: 5z2c). Thr237, Tyr254, Ser277 and Arg150 are shown in stick representation and coloured by element (carbon: bronze; nitrogen: blue; oxygen: red). ADP-H is also shown in stick representation and coloured similarly (except carbon: green; phosphate: orange). The interactions between Thr237 and ADP-H, between Arg150 and ADP-H, and between Tyr254 and Ser277 are shown by black broken lines. (*b,c*) ALPK1 KO cells were transfected with plasmids encoding WT ALPK1 or the indicated ALPK1 mutants and 24 h later incubated with (blue bars) or without (grey bars) 5 μM ADP-H. The activation of NF-κB/AP-1-dependent gene transcription was then measured after a further 24 h as in figure 2 (see §2). Results are shown as the mean of an experiment performed in triplicate, where individual values are denoted by filled circles and the error bars indicate plus and minus one standard error of the mean. Similar results were obtained in two additional, independent experiments. (*d*) FLAG-tagged WT ALPK1 (WT, grey bars), ALPK1[Thr237Ala] (T237A, blue bars), ALPK1[Tyr254Phe] (Y254F, yellow bars) and ALPK1[Ser277Ala] (S277A, pink bars) were immunoprecipitated from cell extracts and assayed and plotted as in figure 3. Two independent experiments were performed, each in duplicate. The results from each experiment were averaged and represented by filled circles. The bar heights reflect the mean of these two values.

We compared the positions of Ser277, Tyr254 and Thr237 within the crystallographic structure of the ADP-heptose binding domain of ALPK1, which has been solved in the ADP-heptose-bound state (PDB: 5z2c) [1]. Thr237 is situated within the ADP-heptose binding site itself, where it forms a hydrogen bond with a hydroxyl group on the sugar moiety of ADP-heptose (figure 4*a*). In contrast, Tyr254 is located within an α-helical region just outside the ADP-heptose binding site where, interestingly, it is in close proximity to Ser277 on an adjacent α-helix with which it forms a hydrogen bond (figure 4*a*). These observations raised the question of whether ALPK1[Ser277Phe] loses its specificity for ADP-heptose and allows activation by human nucleotide sugars due to the loss of the hydrogen bond between Tyr254 and the hydroxyl side chain of Ser277, or due to the mutation of Ser277 to an amino acid with a large hydrophobic side chain (Phe). To investigate this question, we made the Tyr254Phe and Ser277Ala variants to prevent hydrogen bond formation between Tyr254 and Ser277 without affecting the size of these side chains significantly. We found that both of these variants behaved like WT ALPK1 in transfected cells (figure 4*b*), indicating that it is the bulky hydrophobic side chain introduced by the Ser277Phe mutation that is responsible for modifying the specificity of the ADP-heptose binding site. The ALPK1[Thr237Met] variant disrupts the hydrogen bond formed between the hydroxyl side chain of Thr237 and a hydroxyl group in ADP-heptose. To probe the importance of this interaction in maintaining the integrity of the ADP-heptose binding site, we changed Thr237 to Ala, which has a smaller side chain than Met. Interestingly, the ALPK1[Thr237Ala] mutant displayed even higher ADP-heptose-independent activity than ALPK1[Thr237Met] in transfected cells (figure 4*c*). Finally, we carried out cell-free ALPK1 assays on these mutants. Consistent with the cell transfection experiments, the Tyr254Phe and Ser277Ala mutants, like WT ALPK1, were not activated by UDP-α-d-mannose or ADP-ᴅ-ribose (figure 4*d*), while the Thr237Ala mutant, like the Thr237Met mutant, was activated by UDP-α-d-mannose and ADP-d-ribose. Moreover, like the Ser277Phe mutant, but unlike the ALPK1[Thr237Met] mutant, the ALPK1[Thr237Ala] mutant could also be activated by GDP-mannose (figure 4*d*). These results establish that the hydrogen bond formed between the hydroxyl group of Thr237 and ADP-heptose in WT ALPK1 has an important ‘gatekeeping’ function in preventing interaction with human metabolites. Patients with ROSAH syndrome caused by a Thr237Ala variant have not been identified.

The finding that the ALPK1 variants causing ROSAH syndrome can be activated by human nucleotide sugars distinct from ADP-heptose implies that the conformation of the ADP-heptose binding pocket is altered significantly from that of WT ALPK1. It should therefore be possible to exploit this difference to develop small-molecule drugs that prevent activation of the ALPK1 variants causing ROSAH syndrome without affecting WT ALPK1. Since ROSAH syndrome shows an autosomal dominant inheritance pattern with patients expressing both the WT and a variant form of ALPK1, such drugs should not impair the bacterial defence mechanism conferred by WT ALPK1 and, therefore, not impair patient susceptibility to bacterial pathogens.

## Data Availability

All study data have been included in the main article and in the electronic supplementary material, available online [14].
